# Application prospective of nanoprobes with MRI and FI dual-modality imaging on breast cancer stem cells in tumor

**DOI:** 10.1186/s12951-016-0195-8

**Published:** 2016-06-23

**Authors:** Hetao Chen, Yu Wang, Tong Wang, Dongxing Shi, Zengrong Sun, Chunhui Xia, Baiqi Wang

**Affiliations:** Department of Occupational and Environmental Health, School of Public Health, Tianjin Medical University, No. 22, Qixiangtai Road, Heping District, Tianjin, 300070 China; Department of Chemistry, Qiqihaer Medical College, Qiqihaer, 161006 Heilongjiang Province China

**Keywords:** Breast cancer, Cancer stem cells, Magnetic resonance, Fluorescence, Imaging

## Abstract

Breast cancer (BC) is a serious disease to threat lives of women. Numerous studies have proved that BC originates from cancer stem cells (CSCs). But at present, no one approach can quickly and simply identify breast cancer stem cells (BCSCs) in solid tumor. Nanotechnology is probably able to realize this goal. But in study process, scientists find it seems that nanomaterials with one modality, such as magnetic resonance imaging (MRI) or fluorescence imaging (FI), have their own advantages and drawbacks. They cannot meet practical requirements in clinic. The nanoprobe combined MRI with FI modality is a promising tool to accurately detect desired cells with low amount in tissue. In this work, we briefly describe the MRI and FI development history, analyze advantages and disadvantages of nanomaterials with single modality in cancer cell detection. Then the application development of nanomaterials with dual-modality in cancer field is discussed. Finally, the obstacles and prospective of dual-modal nanoparticles in detection field of BCSCs are also pointed out in order to speed up clinical applications of nanoprobes.

BC is serious malignant tumor to threat lives of women. The new cases reach 1,300,000 annually worldwide. Despite identification and treatment technologies have achieved great progress, BC is still the second largest cause of tumor-related deaths of women [1]. BC does not have any symptom at early stage. The unformed nodules are too small to be perceived in clinic exams. Usually when patients find tangible lumps, metastasis can happen in the whole body and tissue-focused therapy is highly likely to fail [2, 3]. Therefore, the diagnosis and cure of BC at early stage is highly necessary to decrease mortality and improve the quality of the lives of patients.

## Breast cancer stem cells

**Fig. 1:**
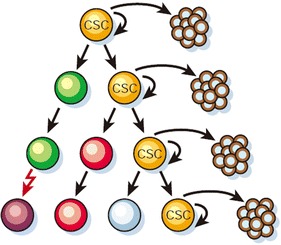
Most cancer cells have only limited proliferative potential, only CSCs (*yellow*) have the ability to proliferate extensively and form new tumors [5] [Adapted by permission from Macmillan Publishers Ltd: [Nature] (Ref.5), copyright (2001)]

With accumulating basic and clinical knowledge, the treatment technologies of BC are continuously created, and survival period of patients is gradually prolonged. Although the 5-year overall survival of BC reaches 91 % [1], it is still unavoidable that about 30 % patients happen recurrence and metastasis [4]. In recent years, scientists find that BC is a typical disease of stem cells, whose recurrence and metastasis are close related to the CSCs (Fig. 1) [5].

CSC hypothesis proposes that one population with rare quantity have the capability of self-renewal, proliferation and high resistance to chemotherapy drugs [6]. The CSCs hypothesis supplies a new clue for diagnosis and therapy of cancers. In 1997, Bonnet et al. [7] firstly indentified a common immunophenotype of leukemic stem cells with self-renewal potential. Therefore, CSC existence was firstly proved. In 2003, AI-Hajj successfully isolated BCSCs from human BC cell line. One thousand of these cells were sufficient to generate tumors when xenotransplanted into NOS/SCID mice, although around fifty thousand were needed in the unsorted population. That work demonstrated that BC originated from BCSCs [8].

The proliferation of BCSCs is disordered and out of programming, and BCSCs lack differentiation and mature capability. Moreover, BCSCs can accumulate replication error, furthermore, lead to tumor occurrence. BCSCs can also escape chemotherapy, achieve drug resistance ability through mutation, and accelerate self-renewal process after drug therapy [9]. These factors promote breast cancer recurrence. In addition, activity of BCSCs can enhance and ratio of side population will increase after radiation treatment. Furthermore, BC has capability to resist radiotherapy [10, 11]. Although BCSCs play critical roles in occurrence, development and recurrence of BC, the amount of BCSCs in tumor tissue is less than 2 % [12]. Therefore, isolation, detection and labeling BCSCs are difficult. But they are still study focuses in BC field at present.

The isolation is foundation and key of BCSCs investigation. At present, the approaches of BCSCs isolation include surface marker sorting, aldehyde dehydrogenase activity assay, flow cytometry sorting side population, etc. [8–15]. Almost all of the described methods are based on optical change before and after substrates interacting with antibodies. Then BCSCs are isolated by flow cytometry. These methods are able to rapidly isolate, purify BCSCs. They push forward the advancement of BCSCs research, and enrich understanding of BCSCs for scientists [16, 17]. However, the former approaches are mainly well applied in cell line level; and they have difficulties to track BCSCs in solid tumors in vivo. In this side, fluorescence quantum dot (QD) probes have intrinsic advantages in detecting cancer cells.

## Fluorescent quantum dot probes staining BC and other cells

The QD probes based on semiconductor quantum dots have obvious advantages. They have broad excitation band, narrow and symmetrical fluorescence peak, tunable fluorescence wavelength with adjusting diameter and components of nanoparticles and strong anti-photobleaching capability, comparing with traditional organic dyes. The fluorescence intensity and stability of single QDs are 20 and 100–200 times higher than that of single organic fluorescence molecule, in respectively [18]. In 1998, Alivisatos and Nie groups published papers about QDs applications in biological systems in Nature magazine at the same time, which marked era arrival of fluorescence QD labeling biological molecules [19, 20].

**Fig. 2:**
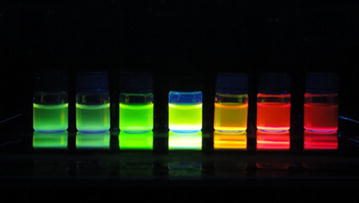
Emission colors of QDs excited by UV

**Fig. 3:**
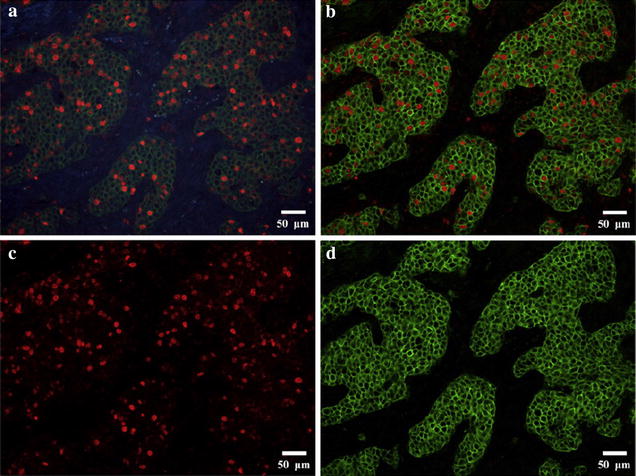
QD-based double-color in situ fluorescent imaging for Ki67 andHER2 in BC. QD-based double-color images for Ki67 andHER2, Ki67was expressed as clear red fluorescence, HER2 as bright green fluorescence (**a**); the spectral images of Ki67 and HER2 co-expressions were obtained by CRi Nuance multispectral imaging system, which could unmix the images into single color images (**b**); the single *red fluorescent* image representing Ki67 at the emitting wavelength of 655 nm (**c**); and the single *green fluorescent* representing HER2 at the emitting wavelength of 525 nm (**d**). 200× , *scale bar* = 50 μm [28]

QD probes can specially label biomarkers on tumor cell surface and accurately label subtle subcellular structure. Moreover, two or more different QDs can be excited by sole light source (Fig. 2). Wu et al. linked QDs with immunoglobulin G (IgG) and streptavidin to label breast cancer marker Her2 on the surface of fixed and live cancer cells. The QD probes could specifically label the desired targets and were brighter and considerably more photostable than comparable organic dyes. Their study demonstrated that QD probes could be very effective in cellular imaging and offer substantial advantages over organic dyes in multiplex target detection [21]. Gao and O`Regan et al. conjugated Her2, ER, PR, mTOR and EGFR with QDs to obtain nanoprobes. Their QD probes could not only detect tumor biomarkers in both cultured human BC cells and on single paraffin embedded clinical tissue sections, but also quantify ER, PR and Her2 receptors. Their study suggested that QD probes were well suited for molecular profiling of tumor biomarkers in vitro [22]. Pang and Li et al. even used QDs immunofluorescence technology to quantify HER2 expression in BC [23]. Liu and co-workers used QD probes to explore basic scientific problem, BC invasion. They obtained exciting results [24]. Many groups, including our research team, have carried out relevant studies and the results were interesting [25–31] (Fig. 3).

**Fig. 4:**
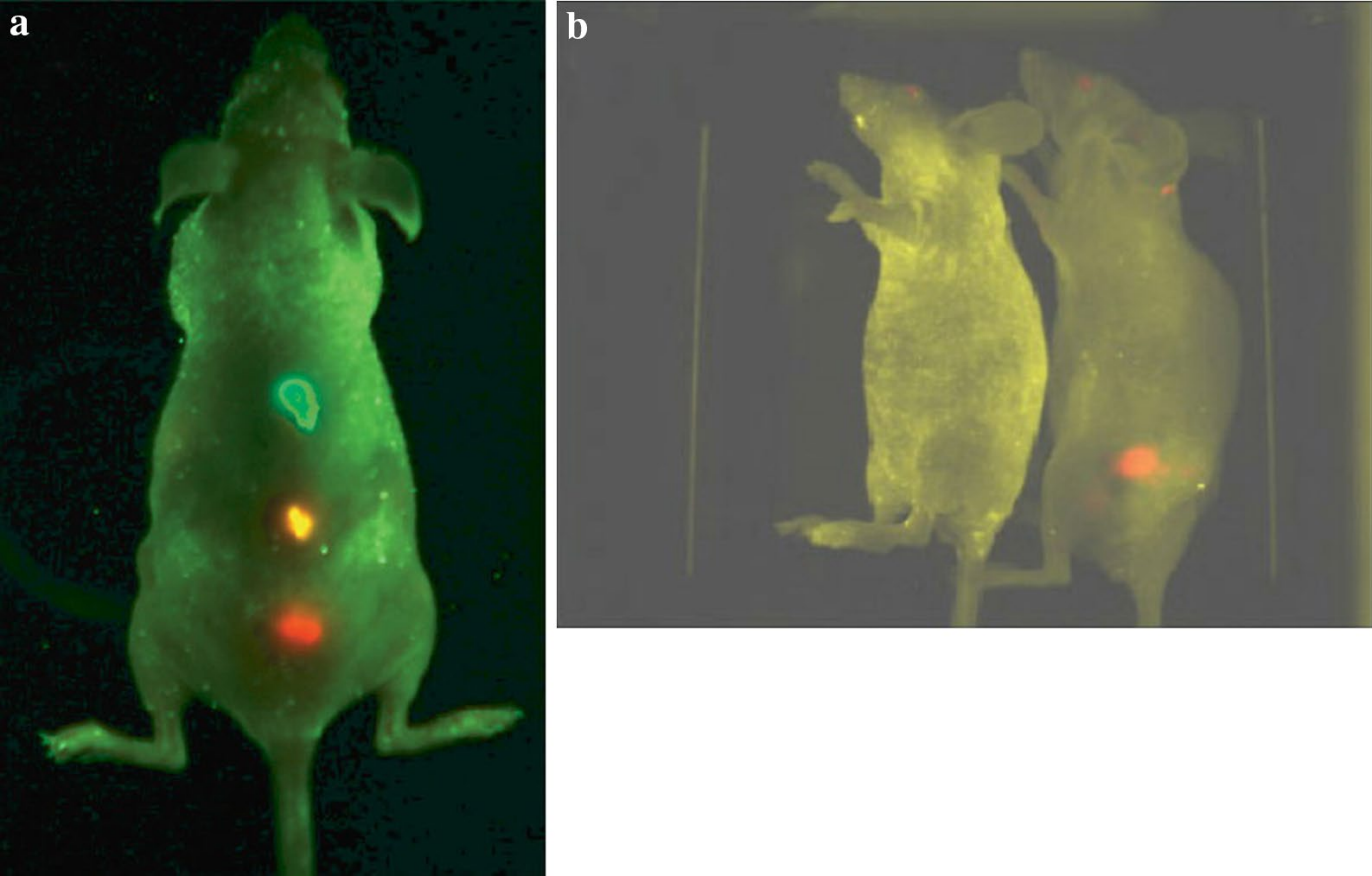
**a** In vivo simultaneous imaging of multicolor QD-encoded microbeads injected into a live mouse (**b**) Molecular targeting and in vivo imaging of a prostate tumor in mouse using a QD–antibody conjugate (*red*) [32]

QDs have excellent optical properties, therefore, they have great potential to be applied in tumor imaging field as fluorescence probes in vivo. Gao and co-workers successfully attempted. They encapsulated luminescent QDs with ABC triblock copolymer and linked this amphiphilic polymer to tumor-targeting ligands and drug-delivery functionalities. In vivo targeting studies of human prostate cancer demonstrate that QD probes could accumulate at tumor sites through passive and active targeting effects. The QD probes displayed sensitive and multicolor fluorescence imaging of cancer cells under in vivo conditions (Fig. 4) [32]. Their study and results of other groups demonstrated that QD probes could probably be used for ultrasensitive and multiplexed imaging of molecular targets in vivo [33, 34]. Although QD probes have their own advantages, it also has some unavoidable disadvantages in vivo detection cells with low amount of quantity. The wavelength of emission photon of QD is in visible range, which is also emission band of tissue autofluorescence. Moreover, the fluorescence of QD has low spatial resolution in organ analysis. These drawbacks can effectively be compensated by the magnetic resonance imaging (MRI).

## MRI labeling BC and other cells

MRI has rapidly developed and widely used in biology and medicine field since it was firstly applied in human disease diagnosis in 1973 [35]. MRI is based on magnetic resonance signal change of water nuclei of hydrogen atoms under the interaction of an external magnetic field [36]. MRI has its own advantages in tumor detection at early stage in vivo.

The contrast agent is an important part of MRI technology, and it is estimated that more than 35 % clinical MRI diagnosis must use contrast agent [37]. At present, gadopentetatedimeglumine (DTPA-Gd) is most commonly used in clinic. Although the proton T1 relaxation time decreases and the clarity of MRI improves after DTPA-Gd intravenous injection, the contrast agent belongs to small molecular compound and its price is relatively expensive. Moreover, the contrast agent does not have tissue or organ targeting effect, and its retention time is short. These disadvantages limit its further applications in clinical practice [38].

**Fig. 5:**
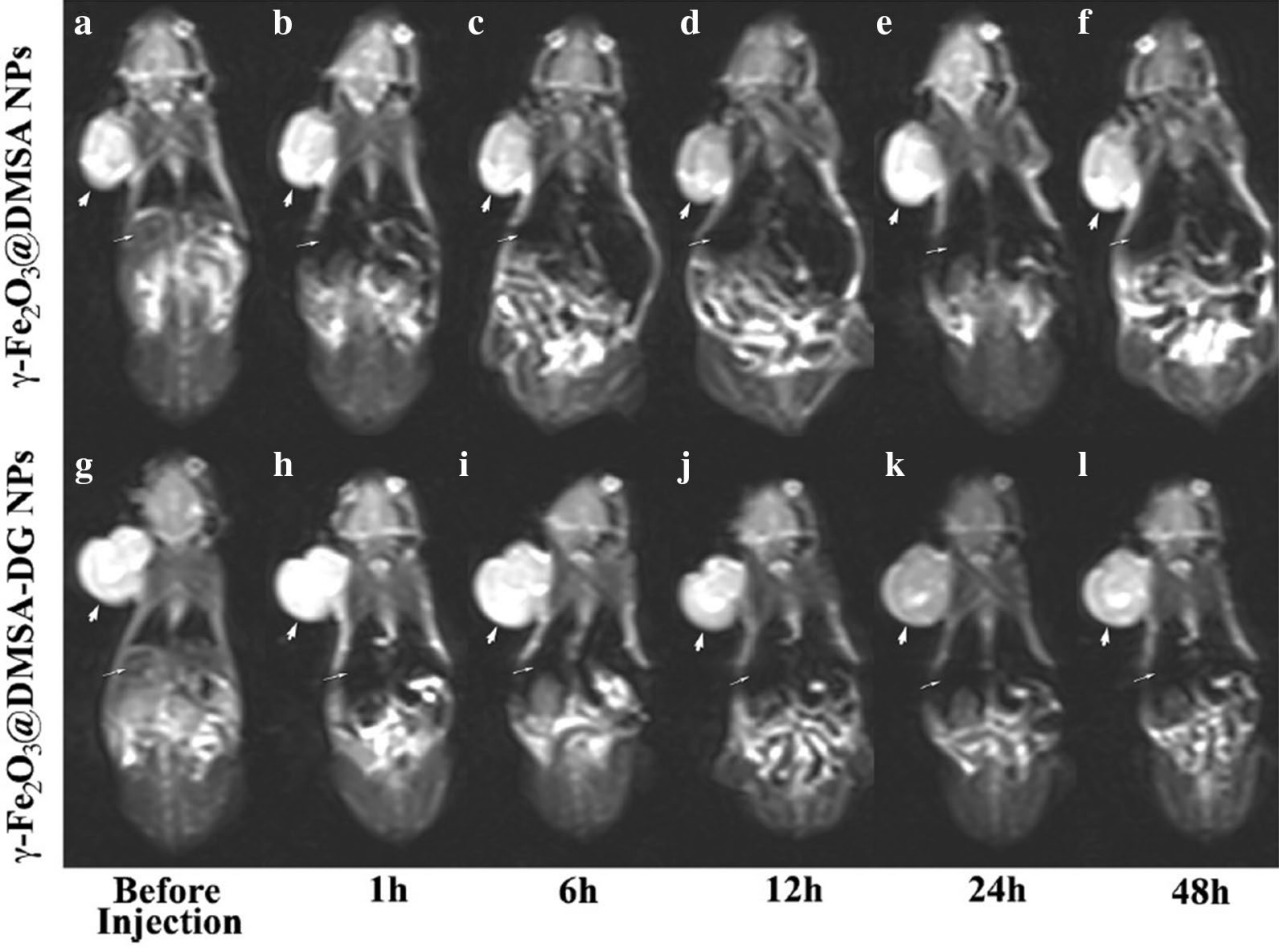
1.5-T MRI turbo-spin-echo-T2-weighted (5500/100) dynamic imaging of human MDA- MB-231 breast cancer xenografts. **a**–**f** Injection of γ-Fe_2_O_3_@DMSA NPs showed that the tumor (*thick white arrows*) signal intensity decreased at 12 h which returned to basal levels by 24 h. **g**–**l** Injection of γ-Fe_2_O_3_@DMSA-DG NPs showed that the tumor signal intensity decreased between 12 and 48 h, with the most hypointensity observed at 24 h. The signal intensity in the liver (*thin white arrows*) significantly decreased after injection of γ-Fe_2_O_3_@DMSA NPs or γ-Fe_2_O_3_@DMSA-DG NPs [48]

With nanotechnology development, scientists began to explore application possibility of magnetic nanomaterials as imaging contrast agent in MRI [39]. Up to now, most studies focus on superparamagnetic iron oxide (SPION). The core crystal structure of SPION is Fe_2_O_3_, Fe_3_O_4_ or their composite. The SPION surface is modified with some functional molecules, such as dextran, citrate or polyethylene glycol, in order to improve stability and biocompatibility of contrast agent [40–42]. SPION has magnetic property. While the size of SPION particles reaches nanometer level, the single magnetic domain can be formed. Therefore, the iron oxide nanoparticles appear superparamagnetic property [43]. Then SPION nanoparticles can further conjugate with biofunctional molecules to fabricate targeting nanoprobes, which are able to detect BC cells and other cells. Salouti and Shayesteh et al. prepared SPION coated with dextran and bombesin to produce a targeting contrast agent (DSPION-BBN) for detection of BC using MRI. They found that DSPION-BBN possessed good diagnostic capability as a contrast agent, with appropriate signal reduction in T_2_^*^-weighted color map MR imaging in mice with BC [44]. On SPION surface, other functional molecules can also be conjugated; furthermore, SPION bioconjugates possess more functions. Zheng and Wang et al. prepared superparamagnetic poly(lactic-co-glycolic acid) (PLGA) microcapsules (Fe_3_O_4_/PLGA) for the application in ultrasound/MRI dual-modality biological imaging of BCs in vitro and in vivo. Their results showed that the bioconjugates had good ultrasound imaging and MRI imaging capability and provided an alternative strategy for highly efficient imaging guided non-invasive BC therapy [45]. Other research groups also obtained similar good results [46–48] (Fig. 5).

SPION has good properties for MR imaging, but it also has some intrinsic drawbacks. In MR imaging, the SPION shortens the T_2_ time, and makes the T2 weighted image darker, thus enhances the contrast. Therefore, MRI signal is weak when few of CSCs in tissue is detected. In addition, a study has demonstrated that the amount of iron oxide consumed by the cells was 10 times over the amount of endogenous iron in detection process. This could directly induce some toxicities and side effects to the cells [49]. Moreover, the endosomes containing particles of iron oxide were particularly sensitive to external magnetic field. The endosomes could be arranged along the outer magnetic field and the bead string structure could be formed in the intracellular [50]. All of these drawbacks influence the quality of MR imaging CSCs.

According to the former analysis, nanomaterials with single imaging modality, whatever is FI or MRI modality, cannot sensitively and accurately label and track few CSCs in solid tumors because of their natural advantages and disadvantages (Table 1). But once the nanoprobes combined with FI and MRI modality, namely FI-MRI dual-modality, are possibly to label and track CSCs in in vivo.

**Table 1:** Advantages and drawbacks of fluorescence and magnetic resonance imaging

	Fluorescence	Magnetic resonance imaging
Spatial resolution	Low	High
Sensitivity	High	Low
Specificity	Weak	T1 imaging is better for anatomic structure; T2 imaging is better for tissue lesions
Penetration depth	Shallow	Deep
Acquisition time	From a few seconds to a few minutes	From several minutes to a few hours
Traumatic	Noninvasion	Noninvasion

## FI-MRI dual-modality nanoprobes indentifying BC and other cells

Up to now, the nanoprobe studies about FI-MRI dual modality for labeling cells have achieved advancement worldwide. Most of the studies were based on nanosized iron oxide to fabricate dual-modality nanoprobes. Shi and Yang group prepared monodisperse silica-coated manganese oxide nanoparticle (NPs) covalently conjugated with Rhodamine B isothiocyanate (RBITC) and folate (FA) on surface. The prepared nanoprobes could specifically target cancer cells overexpressed FA receptors. And the probes were excellent platform for both MRI and FI in various biological systems at the same time [51]. Lee et al. constructed dual-modality nanoprobes based on a Fe_3_O_4_-encapsulated block copolymer conjugating with fluorescent dye Sulforhodamine 101. The nanoprobes could be internalized into BC cells, which were probably used in biomedical diagnosis fields [52]. While Xu and co-workers fabricated an FI-MRI dual-modality imaging nanoprobe based on gadolinium oxide and aptamer-Ag nanoclusters. Using this nanoprobe, MCF-7 BC cells could be effectively tracked by FI and MRI in vitro [53]. Kobayashi et al. used small particle of iron oxide (SPIO) and quantum dot (QD) to dual-label human BC, and tracked BC in the lymphatic system in mice in vivo MRI and FI imaging. Their study demonstrated that nanoprobes with MRI and FI dual-modality was possible to depict marco and early micrometastase with the lymphatic system [54]. Zhang et al. prepared SPION coated with copolymer of chitosan and polyethylene glycol (PEG), labeled with fluorescent dye and conjugated with monoclonal antibody against the neu receptor (NP-neu). The bioconjugates could accurately label breast tumors with MRI and optical dual-modality [55].

**Fig. 6:**
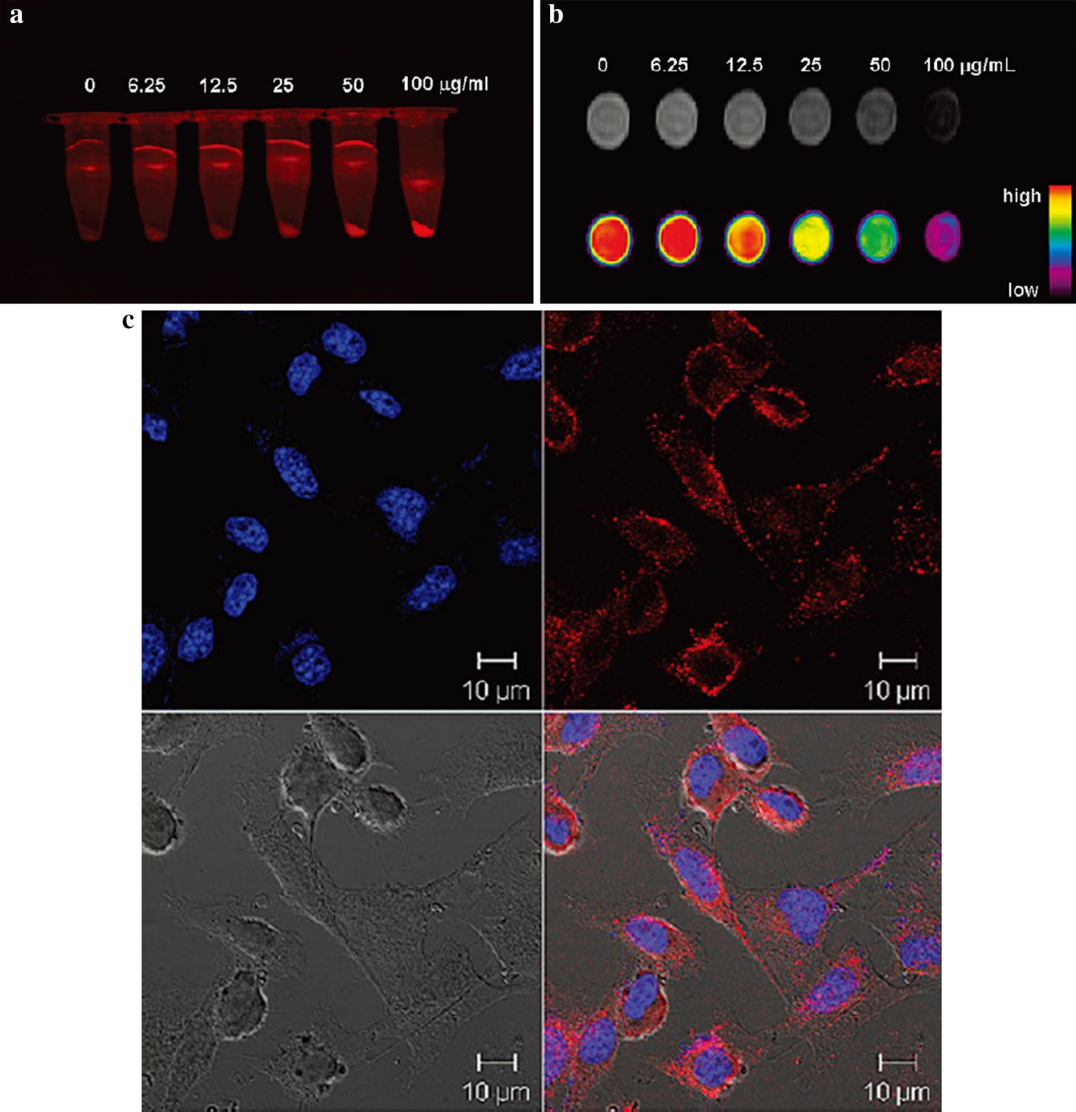
In vitro multimodal imaging with nanoprobes. (**a**) Fluorescence image of cell pellets and (**b**) MR (*upper*) and its color mapped (*lower*) images of dispersed cells in agarose. (**c**) Confocal laser scanning microscopic images [57]. Reprinted with permission from (*J Am Chem Soc.* 2010; **132:** 552–557). Copyright (2010) American Chemical Society

Halas and his assistants constructed nanoshells (NS) by coating a gold shell with a silica epilayer doped with Fe_3_O_4_ and the fluorophore indocyanine green (ICG). The NS enhanced the fluorescence of ICG through efficiently integrating nanoparticles Fe_3_O_4_ into the requisite spacer layer between the metallic shell layer and the ICG fluorophore. The nanocomplexes could well target and image SKBR3 cells [56]. Hyeon and his co-workers synthesized nanoparticles by decorating the surface of mesoporous dye-doped silica nanoparticles with Fe_3_O_4_ nanocrystals loading doxorubicin (DOX). The nanocomplexes could passively target and accumulate at the tumor sites by both T2 MRI and FI. The versatile nanoplatform was a good imaging and drug delivery system for cancer detection and therapy (Fig. 6) [57]. Shin and Cheon fabricated core-satellite nanoparticles through conjugation of Rhodamine-dye-doped silica (DySiO_2_) nanoparticles with water-soluble magnetism engineered iron oxide (MEIO) nanoparticles and HmenB1 antibodies by using proper cross-linkers. The prepared nanoparticles had excellent dual-modal imaging properties for detection of polysialic acids expressed on various cell lines [58]. Other several research groups used dual-modality nanoprobes to detect or analyze cells, the results were very exciting [59–66]. These results indicate that nanoparticles with MRI and FI dual-modality are able to label and track targeting cells in tissue or solid tumors.

**Fig. 7:**
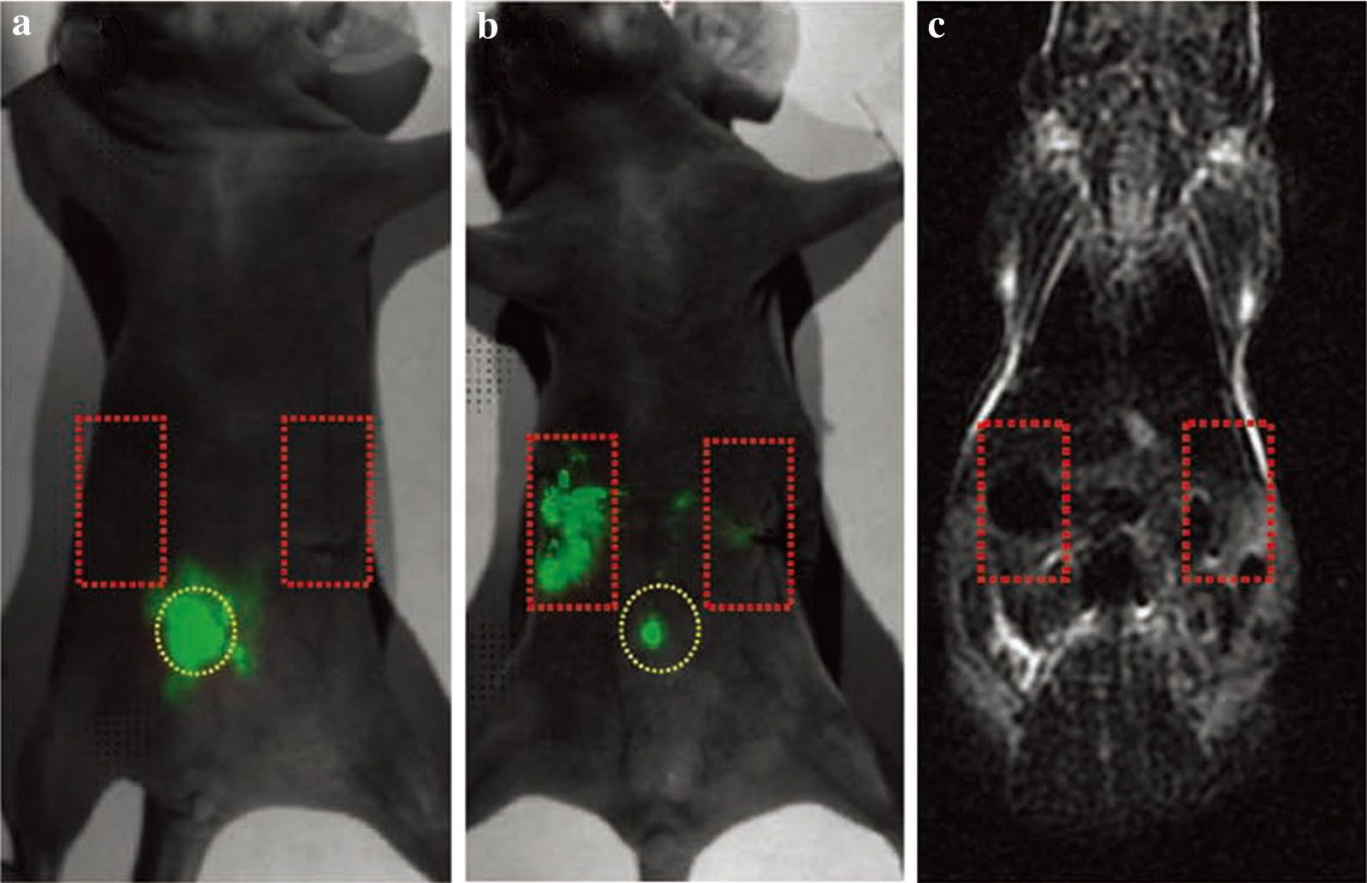
Upconversion fluorescence images of a mouse injected with multifunctional nanoparticles-labeled mMSCs taken right after injection (**a**) and 6 h after injection (**b**) in the presence of a magnetic field. (**c**) In vivo MR image of the same mouse in (**b**) [68]

In detection of stem cells study using dual-modal nanoprobes, several groups have already made progress. Shen et al. constructed nanometer-sized cationic polymersomes loaded with supermagnetic iron oxide nanoparticles and quantum dots. The synthesized cationic polymersomes could act as an effective and safety carrier to transfer image labels into neural stem cells. The monitored cells could be detected up to 6 weeks by MRI and up to 4 weeks by FI [67]. Liu group prepared multifunctional nanoprobes (MFNPs) using upconversion nanoparticles as core, a layer of ultrafine iron oxide nanoparticles as intermediate layer and a gold layer as outer layer. The prepared MFNPs could accurately label mouse mesenchymal stem cells (mMSCs), but they did not influence viability and differentiation ability of mMSCs. In vivo experiments, the nanoprobe exhibited ultrahigh sensitivity. Using upconversion and MRI approaches, MFNP-labeled mMSCs could be well tracked under a magnetic field (Fig. 7) [68].

**Fig. 8:**
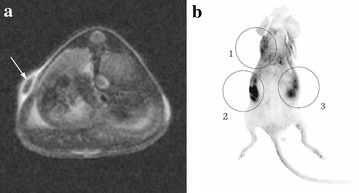
In vivo MR imaging (**a**) and fluorescence imaging (**b**) of nude mouse after subcutaneous injection of labeled and unlabeled human mesenchymal stem cells with MNP@SiO2(RITC)-PEG (1 = 1 × 10^5^ unlabeled human mesenchymal stem cells for control, 2 = 1 × 10^6^ labeled human mesenchymal stem cells, 3 = 1 × 10^5^ labeled human mesenchymal stem cells). Labeled human mesenchymal stem cells are clearly seen as *dark dot* (*arrow*) in subcutaneous layer of nude mouse on axial scan of fast spin echo sequence. Fluorescent signal of subcutaneously injected human mesenchymal stem cells is detected at injection sites of labeled cells. Injection sites of *unlabeled cells* shows autofluorescence-induced artifact [69]

In mesenchymal stem cell detection, Sung and his co-workers prepared magnetic nanoparticles (MNPs) coated with a silica shell, and then rhodamine B siothiocyanate (RITC) was incorporated into the silica shell. Thus, the MNP@SiO_2_(RITC) had a bifunctional property which enables dual modality detection by MRI and optical imaging. The nanoparticles were further modified with PEG groups in order to improve their biocompatibility. The fabricated nanoparticles could accurately label human mesenchymal stem cells (hMSCs) in vitro and in vivo with optical and MRI (Fig. 8) [69]. Park et al. used MRI/FI nanoparticles as transfection agent for gene delivery and cell tracking of hMSCs. The nanoagent did not only display high transfection efficiency in hMSCs, but also exhibit good MRI and FI capability over 14 days [70]. Other research groups used similar bifunctional nanoparticles to label MSCs and similar results were obtained [71–76].

Although these studies are very exciting, but some drawbacks existing in present probes should be perceived. Firstly, optical stability of organic dyes is relatively weak, which can decrease the optical stability of nanoprobes; secondly, the diameter of dual-modality nanoprobes synthesized by polymer composites is relatively larger. This influences penetration capability of nanoprobes among tissue cells and limits nanomaterials further application. In addition, multi-layer assembled nanoprobe has complex structures and is relatively expensive, which is difficult to apply in clinical practice. However, once these drawbacks are overcome, dual-modality or multi-modality nanoprobes will be rapidly applied in detection and therapy fields of BCSCs and other cancer stem cells.

## Application prospective of nanoprobes with MRI-FI dual-modality on detecting BCSCs

MRI-FI Dual-modality nanoprobes are integration of two different imaging probes with single modality. These nanoprobes can be detected under FI and MRI modality at the same time. The nanoprobes with FI-MRI dual-modality do not only overcome drawbacks of single-modality probes, but also compensate multi-drawbacks. The dual-modality nanoprobes can accurately label and track CSCs, and can display the spatial distribution of BCSCs in solid BC tumors. These can afford strong clinical foundation for further studying diagnosis, treatment and recurrence of BC at early stage.

## Abbreviations

BC: breast cancer
CSCs: cancer stem cells
BCSCs: breast cancer stem cells
MRI: magnetic resonance imaging
FI: fluorescence imaging
DTPA-Gd: gadopentetatedimeglumine
SPION: superparamagnetic iron oxide
PLGA: superparamagnetic poly(lactic-co-glycolic acid)
NPs: nanoparticles
RBITC: rhodamine B isothicyanate
FA: folate
QD: quantum dot
PEG: polyethylene glycol
NS: nanoshells
ICG: indocyanine green
DOX: doxorubicin
DySiO_2_: rhodamine-dye-doped silica
MEIO: magenetism engineered iron oxide
MFNPs: multifunctional nanoprobes
mMSCs: mouse mesenchymal stem cells
MNPs: magnetic nanoparticles
hMSCs: human mesenchymal stem cells
